# A Mathematical Model for Thermosensitive Liposomal Delivery of Doxorubicin to Solid Tumour

**DOI:** 10.1155/2013/172529

**Published:** 2013-01-17

**Authors:** Wenbo Zhan, Xiao Yun Xu

**Affiliations:** Department of Chemical Engineering, Imperial College London, South Kensington Campus, London SW7 2AZ, UK

## Abstract

The effectiveness of anticancer treatments is often hampered by the serious side effects owing to toxicity of anticancer drugs and their undesirable uptake by healthy cells *in vivo*. Thermosensitive liposome-mediated drug delivery has been developed as part of research efforts aimed at improving therapeutic efficacy while reducing the associated side effect. Since multiple steps are involved in the transport of drug-loaded liposomes, drug release, and its uptake, mathematical models become an indispensible tool to analyse the transport processes and predict the outcome of anticancer treatment. In this study, a computational model is developed which incorporates the key physical and biochemical processes involved in drug delivery and cellular uptake. The model has been applied to idealized tumour geometry, and comparisons are made between continuous infusion of doxorubicin and thermosensitive liposome-mediated delivery. Results show that thermosensitive liposome-mediated delivery performs better in reducing drug concentration in normal tissues, which may help lower the risk of associated side effects. Compared with direct infusion over a 2-hour period, thermosensitive liposome delivery leads to a much higher peak intracellular concentration of doxorubicin, which may increase cell killing in tumour thereby enhancing the therapeutic effect of the drug.

## 1. Introduction

As a common anticancer drug, doxorubicin is widely used in chemotherapy to treat various types of cancer, such as lymphoma, genitourinary, thyroid, and stomach cancer [1]. By interacting with DNA in cells, doxorubicin can inhibit the process of DNA replication. Because of this mechanism of action, high concentration of doxorubicin in normal tissues can cause serious damage to healthy cells, known as side effects. In clinical therapy, the most serious toxicity is life-threatening cardiomyopathy [2, 3], leading to heart failure. Side effects set a limit to the lifetime dose a patient can receive, which is approximately 550 mg per unit body surface area [1].

In order to improve the therapeutic benefit while reducing toxicity of doxorubicin in normal tissues, various treatment modalities have been developed. Recently, liposome-mediated doxorubicin delivery has been proposed as an alternative to direct intravenous administration. Some animal experiments have shown that liposomal doxorubicin delivery offers better effectiveness of anticancer treatments than bolus injection, but no obvious advantage over continuous infusion was reported [4]. The development of thermosensitive liposomes to enhance the effectiveness of anticancer treatment has been reported in many studies (e.g., [5–8]).

Following administration, the drug-loaded thermosensitive liposome-based nanoparticles are usually small enough to pass through the vasculature wall and then accumulate in the extracellular space in tumour. Localised heating can be performed several hours after drug administration. Upon heating to the phase transition temperature of the thermosensitive liposome, the encapsulated drug can be released from liposomes at a high rate. Some of the released drug may bind with proteins in blood and be cleared up by blood flow, whereas the rest will permeate through the vasculature wall entering the interstitial space. Drug in the interstitium may also bind with proteins present in the interstitial fluid, and be cleared up by the lymphatic system. Because of the concentration gradient at the interface between tumour and normal tissues, drug exchange takes place between these tissues. The extracellular drug may pass through the cell membrane and be taken up by cells. Drug in tumour cells can also be transported back to the extracellular space. Given the many variables related to the properties of tumour, normal tissues, and anticancer drugs, mathematical models are needed to analyse the drug transport processes described above.

Previous numerical studies of liposome-mediated drug delivery have mainly focused on drug uptake by tumour cells with a simplified description of the transport processes involved. Harashima et al. [9, 10] and Tsuchihashi et al. [11] developed mathematical models for nonthermosensitive liposomal drug delivery, without considering the interaction between drug and proteins in blood plasma or interstitial fluid. El-Kareh and Secomb [12] used mathematical models to determine tumour cell uptake of thermosensitive liposome-mediated doxorubicin, but their model was formulated on a simplified tumour cord geometry, without accounting for the influence of blood and lymphatic vessels and the interstitial fluid flow, nor drug binding with proteins. However, each of these components may affect the outcome of anticancer therapy. Experimental results show that doxorubicin can easily bind with proteins [13]. 

In the present study, an improved mathematical model is developed and applied to an idealized geometry consisting of tumour and normal tissues. The model incorporates the key physical and biochemical processes involved, including time-dependent plasma clearance, liposome, and drug transport through the blood and lymphatic vessels, extracellular liposome, and drug transport (convection and diffusion), drug binding with proteins, lymphatic drainage, interactions with the surrounding normal tissues, and drug uptake by tumour cells. Therapeutic effect is evaluated based on the fraction of survival tumour cells by directly solving the pharmacodynamics equation using the predicted intracellular drug concentration. Comparisons are made of the predicted efficacies of direct intravenous administration and thermosensitive liposome-mediated delivery. 

## 2. Mathematical Models

In solid tumours, the size and branching patterns of microvessels could vary considerably depending on the specific tumour type and its growth stage [14]. For a solid tumour at a specific stage, the distribution of blood vessels, lymphatic vessels, and tumour cells are spatially heterogeneous. However, owing to the lack of *in vivo* data on the heterogeneity of tumour vasculature, solid tumours are usually treated as a spatially homogeneous domain [15–18]. If the simulation window is much shorter than the growth rate of the tumour, it would be reasonable to assume that the key modelling parameters do not change with time in the simulation. The mathematical equations governing the physical and physiological processes of the liposome and drug transport as well as the pharmacokinetics of the drug are described below.

### 2.1. Interstitial Fluid Transport

#### 2.1.1. Mass Conservation Equation

This is described by

(1) $$\begin{array}{c} \frac{\partial \rho }{\partial t}+\nabla \cdot \left(\rho v\right)=\left(F_{v}-F_{\text{ly}}\right)\rho , \end{array}$$

where *ρ* and **v** are the density and velocity of the interstitial fluid, respectively. *F*_*v*_ is the interstitial fluid loss from the blood vessels per unit volume of tumour tissue, and *F*_ly_ is the fluid absorption rate by the lymphatics per unit volume of tumour tissue. *F*_*v*_ and *F*_ly_ are given by Starling's law

(2) $$\begin{array}{c} F_{v}=K_{v}\frac{S}{V}\left[p_{v}-p_{i}-\sigma_{T}\left(\pi_{v}-\pi_{i}\right)\right], \end{array}$$

where *K*_*v*_ is the hydraulic conductivity of the microvascular wall, *S*/*V* is the surface area of blood vessels per unit volume of tumour tissue, *p*_*v*_ and *p*_*i*_ are the vascular and interstitial fluid pressures, respectively, *σ*_*T*_ represents the average osmotic reflection coefficient for plasma protein, *π*_*v*_ is the osmotic pressure of the plasma, and *π*_*i*_ is that of interstitial fluid.

The lymphatic drainage, *F*_ly_, is related to the pressure difference between the interstitial fluid and lymphatics:

(3) $$\begin{array}{c} F_{\text{ly}}=K_{\text{ly}}\frac{S_{\text{ly}}}{V}\left(p_{i}-p_{\text{ly}}\right), \end{array}$$

where *K*_ly_ is the hydraulic conductivity of the lymphatic wall, *S*_ly_/*V* is the surface area of lymphatic vessels per unit volume of tumour tissue, and *p*_ly_ is the intralymphatic pressure. 

#### 2.1.2. Momentum Conservation Equation

Since the intercapillary distance (33–98 *μ*m [19, 20]) is usually 2-3 orders of magnitude smaller than the length scale for drug transport (approximately 70 mm in this study), it is reasonable to treat the tumour and its surrounding tissues as porous media, for which the Navier-Stokes equations are applicable. By ignoring the gravitational effect, the momentum equation is expressed as

(4) $$\begin{array}{c} \frac{\partial \left(\rho v\right)}{\partial t}+\nabla \cdot \left(\rho vv\right)=-\nabla p_{i}+\nabla \cdot \tau +F, \end{array}$$

where **τ** is the stress tensor which is given by

(5) $$\begin{array}{c} \tau =\mu \left[\nabla v+{\left(\nabla v\right)}^{T}\right]-\frac{2}{3}\mu \left(\nabla \cdot v\right)I, \end{array}$$

where **I** is the unit tensor. The last term in (4), **F**, represents the Darcian resistance to fluid flow through porous media and is given by

(6) $$\begin{array}{c} F=W\mu v+\frac{1}{2}C\rho \left|v\right|v, \end{array}$$

and *W* is a diagonal matrix with all diagonal elements calculated as

(7) $$\begin{array}{c} W=\kappa^{-1}, \end{array}$$

where *μ* is the dynamic viscosity of interstitial fluid, **C** is the prescribed matrix of the inertial loss term, and *κ* is the permeability of the interstitial space. Since the velocity of interstitial fluid is very slow (|**v** | ≪1) [15], the inertial loss term can be neglected when compared to the Darcian resistance. In addition, the interstitial fluid is treated as incompressible with a constant viscosity. Hence, (6) can be reduced to

(8) $$\begin{array}{c} F=W\mu v. \end{array}$$

### 2.2. Drug Transport

Drug transport is described by equations for the free and bound drug concentrations in the interstitial fluid and the intracellular concentration.

#### 2.2.1. Free Doxorubicin Concentration in the Interstitial Fluid (*C*_*fe*_)

This is described by

(9) $$\begin{array}{c} \frac{\partial C_{fe}}{\partial t}+\nabla \cdot \left(C_{fe}v\right)=D_{fe}\nabla^{2}C_{fe}+S_{i}, \end{array}$$

where *D*_*fe*_ is the diffusion coefficient of free doxorubicin. The source term, *S*_*i*_, is the net rate of doxorubicin gained from the surrounding environment, which is given by

(10) $$\begin{array}{c} S_{i}=S_{v}+S_{b}+S_{u}, \end{array}$$

*S*_*v*_, *S*_*b*_, and *S*_*u*_ represent the net doxorubicin gained from the blood/lymphatic vessels, association/dissociation with bound doxorubicin-protein, and influx/efflux from tumour cells, respectively,

(11) $$\begin{array}{c} S_{v}=F_{fp}-F_{fl}, \end{array}$$

where *F*_*fp*_ is the doxorubicin gained from the blood capillaries in tumour and normal tissues, and *F*_*fl*_ is the doxorubicin loss to the lymphatic vessels per unit volume of tissue. Using the pore model [15–17, 21] for transcapillary exchange, *F*_*fp*_ and *F*_*fl*_ can be expressed as

(12) $$\begin{array}{c} F_{fp}=F_{v}\left(1-\sigma_{d}\right)C_{fp}+P_{fe}\frac{S}{V}\left(C_{fp}-C_{fe}\right)\frac{\text{P}{\text{e}}_{f}}{e^{\text{P}{\text{e}}_{f}}-1},\\ F_{fl}=F_{\text{ly}}C_{fe}, \end{array}$$

where *C*_*fp*_ is the concentration of doxorubicin in blood plasma, *σ*_*d*_ is the osmotic reflection coefficient for the drug molecules, and *P*_*fe*_ is the permeability of vasculature wall to free doxorubicin. Pe_*f*_ is the transcapillary Peclet number defined as

(13) $$\begin{array}{c} \text{P}{\text{e}}_{f}=\frac{F_{v}\left(1-\sigma_{d}\right)}{P_{fe}\left(S/V\right)}. \end{array}$$

The net doxorubicin gained due to protein binding and cellular uptake is governed by (14), where *D*_*c*_ is the tumour cell density; *k*_*a*_ and *k*_*d*_ are the doxorubicin-protein binding and dissociation rates, respectively:

(14) $$\begin{array}{c} S_{b}=k_{d}C_{be}-k_{a}C_{fe},\\ S_{u}=D_{c}\varepsilon -D_{c}\zeta . \end{array}$$

#### 2.2.2. Bound-Doxorubicin Concentration in Interstitial Fluid (*C*_*be*_)

This is described by

(15) $$\begin{array}{c} \frac{\partial C_{be}}{\partial t}+\nabla \cdot \left(C_{be}v\right)=D_{be}\nabla^{2}C_{be}+F_{be}-S_{b}, \end{array}$$

where *D*_*be*_ is the diffusion coefficient of the bound doxorubicin-protein. *F*_*be*_ represents the bound doxorubicin crossing the capillary wall into the interstitial fluid, which is given by

(16) $$\begin{array}{c} F_{be}=F_{v}\left(1-\sigma_{d}\right)C_{bp}+P_{be}\frac{S}{V}\left(C_{bp}-C_{be}\right)\frac{\text{P}{\text{e}}_{b}}{e^{\text{P}{\text{e}}_{b}}-1}, \end{array}$$

where *P*_*be*_ is the permeability of vasculature wall to bound doxorubicin, and *C*_*bp*_ is the bound doxorubicin concentration in plasma. The transcapillary Peclet number is

(17) $$\begin{array}{c} \text{P}{\text{e}}_{b}=\frac{F_{v}\left(1-\sigma_{d}\right)}{P_{be}\left(S/V\right)}. \end{array}$$

#### 2.2.3. Intracellular Doxorubicin Concentration (*C*_*i*_)

Because mainly free doxorubicin can pass through the cell membrane and enter the intracellular space [12], the rate of cellular uptake is a function of free doxorubicin concentration in the interstitial fluid:

(18) $$\begin{array}{c} \frac{\partial C_{i}}{\partial t}=\zeta -\varepsilon ,\\ \zeta =V_{\max}\frac{C_{fe}}{C_{fe}+k_{e}\varphi },\\ \varepsilon =V_{\max}\frac{C_{i}}{C_{i}+k_{i}}, \end{array}$$

where *V*_max⁡_ is the rate of transmembrane transport, *ζ* and *ɛ* are cellular uptake and efflux functions, *k*_*e*_ and *k*_*i*_ are constants obtained from experimental data fitting, and *φ* is the volume fraction of extracellular space.

### 2.3. Thermosensitive Liposome-Mediated Drug Transport

Equations describing the transport of liposome-mediated drug include encapsulated drug concentration in the interstitial fluid, and released doxorubicin in plasma and interstitial fluid. Equations for drug transport include those for free drug concentration in plasma and interstitial fluid. Bound drug concentration in plasma and interstitial fluid as well as intracellular concentration are described using the same equations given in the preceding section.

#### 2.3.1. Liposome Encapsulated Drug Concentration in the Interstitial Fluid (*C*_*le*_)

This is described by

(19) $$\begin{array}{c} \frac{\partial C_{le}}{\partial t}+\nabla \cdot \left(C_{le}v\right)=D_{l}\nabla^{2}C_{le}+S_{l}, \end{array}$$

where *D*_*l*_ is the diffusion coefficient of liposome encapsulated drug. The source term *S*_*l*_ is the net rate of liposome encapsulated drug gained from the surrounding environment, which is given by

(20) $$\begin{array}{c} S_{l}=S_{lp}-S_{r}. \end{array}$$

*S*_*lp*_ is the amount of liposome encapsulated drug from plasma. *S*_*r*_ represents released drug in the interstitial fluid:

(21) $$\begin{array}{c} S_{lp}=F_{lp}-F_{ll}, \end{array}$$

where *F*_*lp*_ is the liposome encapsulated doxorubicin gained from the capillaries in tumour and normal tissues, and *F*_*ll*_ is the loss of liposome encapsulated doxorubicin through the lymphatic vessels per unit volume of tissue. Using the pore model for transcapillary exchange, *F*_*lp*_ and *F*_*ll*_ can be expressed as

(22) $$\begin{array}{c} F_{lp}=F_{v}\left(1-\sigma_{l}\right)C_{lp}+P_{l}\frac{S}{V}\left(C_{lp}-C_{le}\right)\frac{\text{P}{\text{e}}_{\text{l}}}{e^{\text{P}{\text{e}}_{\text{l}}}-1},\\ F_{ll}=F_{\text{ly}}C_{le}, \end{array}$$

where *C*_*lp*_ is the concentration of liposome in blood plasma, *σ*_l_ is the osmotic reflection coefficient for the liposome particles, and *P*_l_ is the permeability of vasculature wall to liposome. Pe_l_ is the transcapillary Peclet number defined as

(23) $$\begin{array}{c} \text{P}{\text{e}}_{\text{l}}=\frac{F_{v}\left(1-\sigma_{l}\right)}{P_{l}\left(S/V\right)}. \end{array}$$

The amount of released liposome encapsulated drug in the interstitial fluid, *S*_*r*_, is given by

(24) $$\begin{array}{c} S_{r}=k_{\mathrm{rel}}C_{le}, \end{array}$$

where *k*_*rel*⁡_ is the release rate of liposome.

#### 2.3.2. Free Doxorubicin Concentration in Blood Plasma (*C*_*fp*_)

This is described by

(25) $$\begin{array}{c} \frac{\partial C_{fp}}{\partial t}=S_{r}-\frac{V_{T}}{V_{B}}F_{fp}-\frac{CL_{fp}C_{fp}}{V_{D}}-\left(k_{a}C_{fp}-k_{d}C_{bp}\right), \end{array}$$

where *F*_*fp*_ represents the free doxorubicin crossing the capillary wall into the interstitial fluid. *V*_*T*_ is tumour volume, *V*_*B*_ is plasma volume, and *V*_*D*_ is the volume of distribution, which is a pharmacological theoretical volume that a drug would have to occupy to provide the same concentration as it is currently in blood plasma. *CL*_*fp*_ is the plasma clearance of drug. *k*_*a*_ and *k*_*d*_ are the association and disassociation rates with proteins.

#### 2.3.3. Bound Doxorubicin Concentration in Blood Plasma (*C*_*bp*_)

This is described by

(26) $$\begin{array}{c} \frac{\partial C_{bp}}{\partial t}=\left(k_{a}C_{fp}-k_{d}C_{bp}\right)-\frac{V_{T}}{V_{B}}F_{be}-\frac{CL_{bp}C_{bp}}{V_{D}}, \end{array}$$

where *CL*_*bp*_ is the plasma clearance of bound doxorubicin. 

#### 2.3.4. Free Doxorubicin Concentration in Interstitial Fluid (*C*_*fe*_)

This is described by

(27) $$\begin{array}{c} \frac{\partial C_{fe}}{\partial t}+\nabla \cdot \left(C_{fe}v\right)=D_{fe}\nabla^{2}C_{fe}+S_{f}. \end{array}$$

The source term *S*_*f*_ is the net rate of doxorubicin gained from the surrounding environment, which is given by

(28) $$\begin{array}{c} S_{f}=S_{v}+S_{b}+S_{u}+S_{r}. \end{array}$$

Expressions for the terms on the right hand side have been given previously (see (11)–(14) and (24)).

### 2.4. Pharmacodynamics Model

During anticancer treatment, tumour cell density may change due to cell killing as a result of drug effect, tumour cell proliferation, and physiological degradation. This can be described by a pharmacodynamics model as given below:

(29) $$\begin{array}{c} \frac{dD_{c}}{dt}=-\frac{f_{\max}C_{i}}{EC_{50}+C_{i}}D_{c}+k_{p}D_{c}-k_{g}D_{c}^{2}. \end{array}$$

The first term on the right hand side represents the effect of anticancer drug, where *f*_max⁡_ is the cell-kill rate constant and *EC*_50_ is the drug concentration producing 50% of *f*_max⁡_. *k*_*p*_ and *k*_*g*_ are cell proliferation rate constant and physiologic degradation rate, respectively. In this study, cell proliferation and physiologic degradation are assumed to reach equilibrium at the beginning of each treatment.

### 2.5. Model Geometry

**Figure 1:**
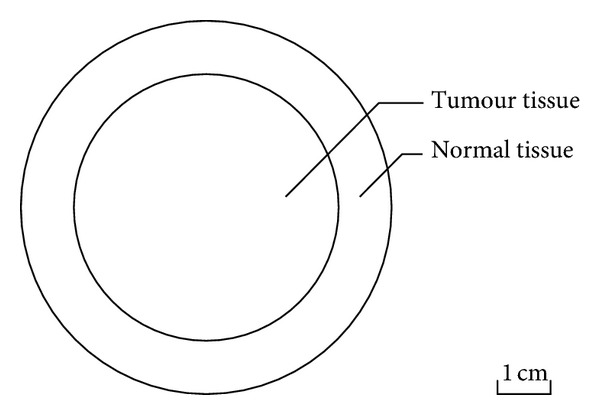
Model geometry.

A 2D idealized model with a realistic tumour size (Figure 1) is used in this study. The tumour is located at the centre, which is surrounded by a layer of normal tissue. The diameter of the tumour is 50 mm, and the thickness of the normal tissue is 10 mm. ANSYS ICEM CFD is used to create the geometry and generate the computational mesh. The final mesh consists of 3922 triangular elements. This is obtained based on mesh independence tests which show that the difference in predicted drug concentration between the adopted mesh and a 10-time finer mesh is less than 3%.

### 2.6. Model Parameters

Since the growth of tumour and normal tissues is ignored, all the geometric and transport parameters used in this study are assumed to be constant. These are summarized in Tables 1, 2, and 3 for parameters related to the tissue, liposome, and doxorubicin, respectively.

#### 2.6.1. Vascular Permeability

Vascular permeability coefficient measures the capacity of a blood vessel (often capillary in tumour) wall to allow for the flow of substances, typically nutrients or pharmaceutical agents in and out of the vasculature. The permeability of polyethylene glycol coated liposomes of 100 nm through tumour capillaries was measured at 37°C by Yuan et al. [23] and Wu et al. [24] as 2.0 × 10^−10^ and 3.42 ± 0.78 × 10^−9^ m/s, respectively. In normal granulation tissues permeability of the same liposomes was 0.8 − 0.9 × 10^−9^ m/s at the same temperature. Wu et al. [26] also measured the permeability of albumin (corresponding to albumin-bound doxorubicin) in tumour and granulation tissues at 37°C and obtained the values of 7.8 ± 1.2 × 10^−9^ m/s and 2.5 ± 0.8 × 10^−9^ m/s, respectively. The mean values of the above measurements are adopted in this study. 

Gaber et al. [5] noticed a 76-fold increase in the liposome extracellular concentration on 45°C heating. The permeability to liposome at 42°C can be estimated by interpolation, which gives a 71-folder increase. Dalmark and Storm [40] measured the permeability of free doxorubicin at various temperatures, and their results showed that the permeability to doxorubicin at 42°C was 2.56-time higher at 37°C. Hence, temperature-dependent vascular permeability for both liposome and doxorubicin is adopted to allow for enhanced permeability at hyperthermia.

#### 2.6.2. Reflection Coefficient

The reflection coefficient determines the efficiency of the oncotic pressure gradient in driving transport across the vascular wall. It is related to the sizes of drug and pores on the vasculature wall [41]. For the same drug, this parameter may vary in different types of tissues [42, 43]. Wolf et al. [32] measured the reflection coefficient for albumin and found this to be 0.82 ± 0.08. The sizes of albumin and liposome are 3.5 nm and 100 nm, respectively. The reflection coefficient for liposome is estimated to be greater than 0.90; hence it is assumed to be 0.95 in this study.

Because the size of liposome is much larger than the pore size on the vasculature wall in normal tissues (24–60 nm in diameter [44]), the reflection coefficient in normal tissue is assumed to be 1.0.

#### 2.6.3. Liposome Release Rate (*k*_*rel*⁡_)

**Figure 2:**
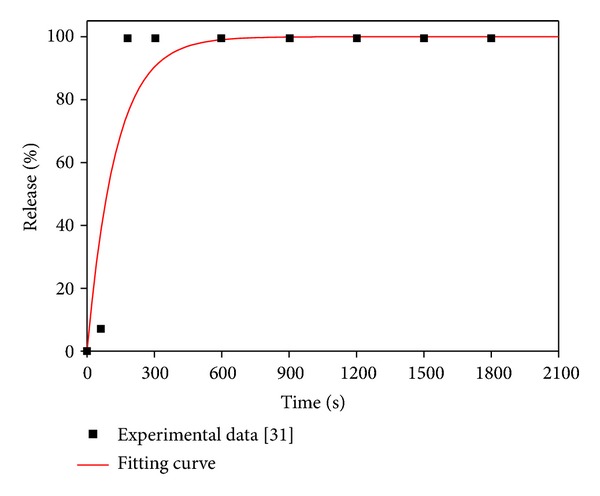
Liposome release rate at 42°C.

Thermosensitive liposome is designed to release its contents rapidly on heating [6]. The release rate varies according to the composition of liposome, its preparation procedure, and heating temperature [45]. The relation between percentage release and exposure time is found to follow the first-order kinetics expressed as [46]

(30) $$\begin{array}{c} \%R\left(t\right)=R_{c}\left(1-e^{-k_{\mathrm{rel}}t}\right), \end{array}$$

where %*R*(*t*) is the percentage of drug released at exposure time *t*; *R*_*c*_ is the total percentage of drug released at a given heating temperature. This equation is used to fit the experimental data obtained at 42°C [45]. From the best fitting curve (shown in Figure 2) obtained by using nonlinear least-squares method, the release rate is found to be 0.0078. At normal physiological temperature of 37°C, there should be no release; hence the release rate at 37°C is assumed to be zero.

#### 2.6.4. Plasma Pharmacokinetics

(1)* Direct Continuous Infusion*. The doxorubicin concentration in blood plasma is modelled as an exponential decay function of time. The form of equation depends on the infusion mode. For continuous infusion, a triexponential decay function is assumed based on the plasma pharmacokinetics of doxorubicin:

(31) Cv=DT[(Aα(1−e−αt)+Bβ(1−e−βt)+Cγ(1−e−γt))]                    (t<T),  Cv=DT[Aα(eαT−1)e−αt+Bβ(eβT−1)e−βt       +Cγ(eγt−1)e−γt] (t≥T),

where *D* is the dose of doxorubicin and *T* is the infusion duration. *A*, *B*, and *C* are compartment parameters and *α*, *β*, *γ* are compartment clearance rates.

Free doxorubicin in plasma can easily bind with proteins, such as albumin. Greene et al. [13] found that 74%–82% is present in the form of bound doxorubicin, and the percentage is independent of doxorubicin and albumin concentrations. Hence for direct infusion, the free (*C*_*fp*_) and bound (*C*_*bp*_) doxorubicin in plasma are given by

(32) $$\begin{array}{c} C_{fp}=\left(1-s\right)C_{v};\;\;C_{bp}=sC_{v}, \end{array}$$

where *s* is the percentage of bound doxorubicin, which is 0.75 in this study.

(2)* Thermosensitive Liposome-Mediated Drug Release*. The liposome encapsulated doxorubicin concentration in blood plasma is found to follow a 2-exponential decaying function of time [13], written as

(33) $$\begin{array}{c} C_{lp}=A_{1}e^{-k_{1}t}+A_{2}e^{-k_{2}t}, \end{array}$$

where *A*_1_ and *A*_2_ are compartment parameters, and *k*_1_ and *k*_2_ are compartment clearance rates.

### 2.7. Boundary Conditions

Because the time scale for the simulation is assumed to be short enough to ignore the growth of tumour and normal tissues, the interface between the tumour and normal tissue as well as the outer surface of normal tissue are fixed. The interface between the tumour and normal tissues is treated as an internal boundary where all variables are continuous. The relative pressure at the outer surface of normal tissues is assumed to be constant at 0 Pa, where zero flux of drug is also specified.

### 2.8. Numerical Methods

The mathematical models described above are implemented into ANSYS FLUENT, which is a finite volume based computational fluid dynamics (CFD) code. Mass transfer equations describing the transport of drugs are coded by using the User Defined Scalar (known as UDS). These equations are solved in conjunction with the continuity and momentum equations using numerical algorithms available in FLUENT. Spatial discretisation is performed by employing the second order UPWIND scheme, while pressure-velocity coupling is achieved by the SIMPLEC algorithm. The absolute criteria for residual tolerances for solutions of the Navier-Stokes equations and the drug transport equations are 1 × 10^−5^ and 1 × 10^−8^, respectively. The equations for the interstitial fluid flow are solved first to obtain a steady-state solution in the entire tumour and its surrounding normal tissues. The obtained pressure and velocity fields are then applied to the equations for drug transport. The second-order implicit backward Euler scheme is used for temporal discretisation, and a fixed time step size of 10 seconds is chosen, which is obtained after time-step sensitivity tests.

## 3. Results and Discussion

The microenvironment in tumour and normal tissues plays an important role in determining the efficiency of liposome and drug transport. The interstitial fluid pressure (IFP) determines the drug exchange between interstitial fluid and blood plasma, as well as tumour and normal tissues. The mean IFP predicted in the tumour region is 1533 Pa, which is almost identical to the value reported by Baxter and Jain [15]. The mean IFP in the normal tissue is 41 Pa.

**Figure 3:**
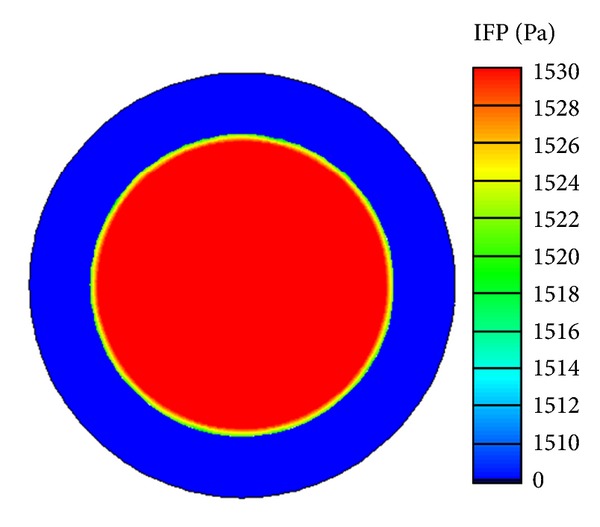
Interstitial fluid pressure distribution in tumour and normal tissues.

The spatial distribution of IFP in tumour and normal tissues is shown in Figure 3. It is clear that pressures in the tumour and normal tissues are at different levels, and a thin layer of steep pressure gradient exists at the interface between the two regions.

**Figure 4:**
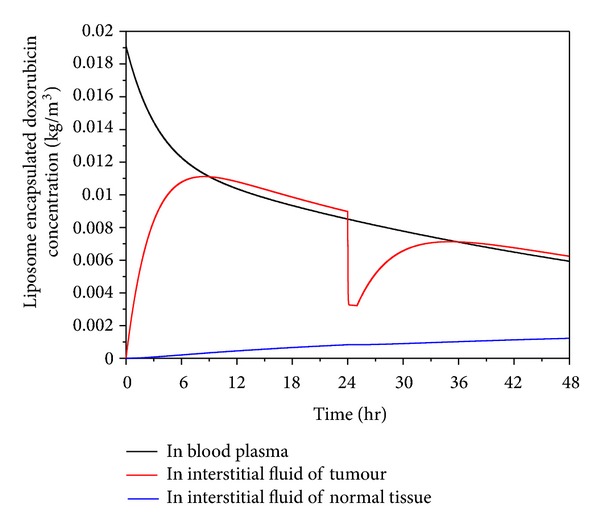
Liposome concentration in plasma and interstitial fluid as a function of time after start of treatment (dose = 50 mg/m^2^).

Liposome encapsulated doxorubicin concentration is a key parameter that determines the doxorubicin concentration in tumour cells. Shown in Figure 4 are the predicted time courses of liposome encapsulated doxorubicin concentrations in blood plasma and interstitial space in tumour and normal tissues, for a total doxorubicin dose of 50 mg/m^2^ encapsulated in thermosensitive liposomes. 

Liposome encapsulated doxorubicin is administrated into blood in a very short duration, and its concentration in plasma decreases following an exponential decay function of time during the entire treatment period [13]. Its concentration in tumour increases rapidly in the initial stage after administration. This is because at this stage, the concentration in plasma is much higher than that in tumour, providing the driving force for liposome to pass through the vasculature wall and accumulate in tumour. The concentration in tumour reaches its peak when the concentration in tumour interstitial fluid and plasma reaches an equilibrium state. Upon heating at 24 hours after administration (heating lasts for 1 hour), doxorubicin is rapidly released from liposome, resulting in a sharp fall in the concentration of liposome encapsulated doxorubicin in the interstitial fluid of tumour, followed by a steady increase after heating ceases at 25 hour.

**Figure 5:**
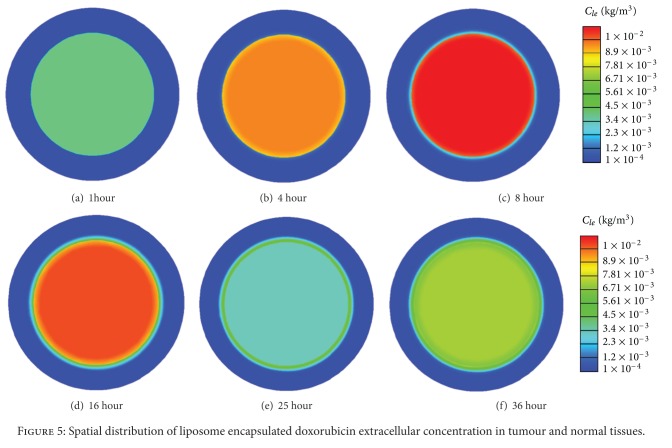
Spatial distribution of liposome encapsulated doxorubicin extracellular concentration in tumour and normal tissues.

Since the size of liposome is too large to pass through the vasculature wall in normal tissues [44, 45], liposome encapsulated doxorubicin enters normal tissues by diffusion and convection from tumour, which can be seen clearly in Figure 5. This is the reason why the liposome encapsulated doxorubicin concentration in normal tissues increases slowly over time and stays at a very low level during the simulation time.

**Figure 6:**
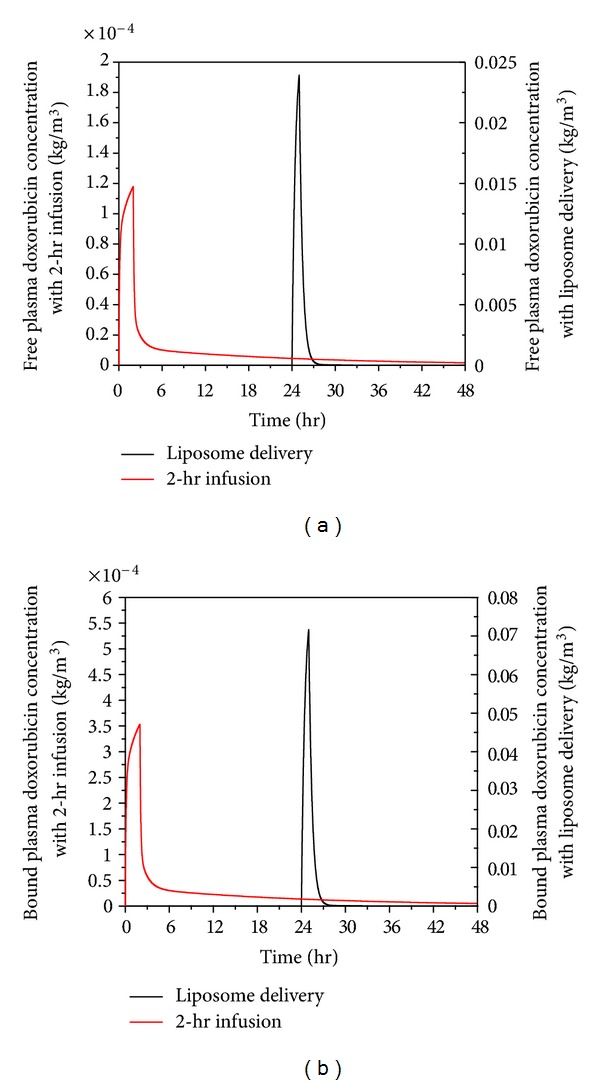
Spatial mean free (a) and bound (b) doxorubicin plasma concentration in tumour as a function of time under liposome delivery and 2-hr infusion of nonencapsulated doxorubicin (dose = 50 mg/m^2^).

There is evidence for rapid and significant binding between free doxorubicin and proteins in plasma [12, 22]. Predicted free and bound doxorubicin concentrations in plasma for thermosensitive liposome delivery and 2-hour infusion of nonencapsulated doxorubicin are compared in Figure 6. Results show that 75% doxorubicin binds with proteins, which is consistent with the experimental data of Greene et al. [13].

For direct infusion of nonencapsulated doxorubicin, the infusion duration is 2 hours as recommended in the literature [12], and the total dose is 50 mg/m^2^. The free and bound doxorubicin concentrations increase rapidly during the initial period following drug administration. For thermosensitive liposome delivery, the doxorubicin concentration remains at zero in the first 24 hours, since no doxorubicin is released from liposome before heating is applied. Upon heating to mild hyperthermia at 24 hours which lasts for one hour, doxorubicin is rapidly released from liposome causing much higher concentration in plasma. Because the temperature of tumour falls back to 37°C immediately after heating is stopped and assuming that encapsulated doxorubicin remains trapped within the core of liposome, the concentration declines rapidly to a low level. Although the concentration with both modes of administration drops to a low level after the infusions ends, 2-hour continuous infusion of nonencapsulated doxorubicin gives a slightly higher concentration over time.

**Figure 7:**
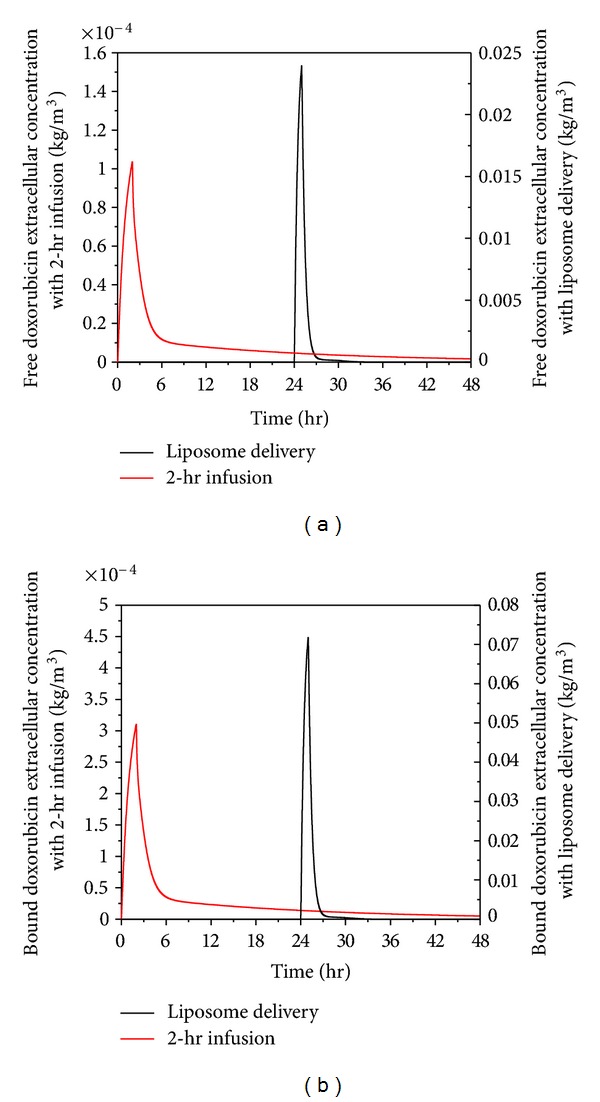
Spatial mean free and bound doxorubicin extracellular concentration in tumour as a function of time under thermosensitive liposome delivery and 2-hour infusion (dose = 50 mg/m^2^).

**Figure 8:**
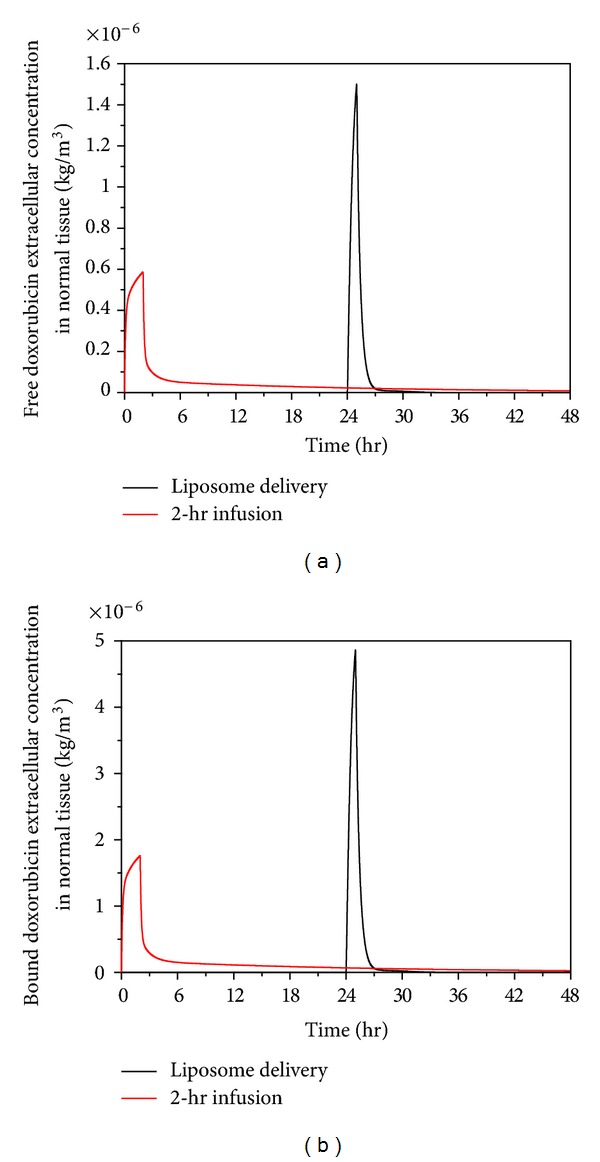
Spatial mean free and bound doxorubicin extracellular concentration in normal tissue as a function of time under thermosensitive liposome delivery and 2-hour infusion of nonencapsulated doxorubicin (dose = 50 mg/m^2^).

Free and bound extracellular concentrations of doxorubicin in tumour and normal tissues are shown in Figures 7 and 8, respectively. Comparing the extracellular concentrations in these two figures with the plasma concentration in Figure 6, they all seem to follow the same trend. This means that plasma concentration has a direct influence on the extracellular concentration of both free and bound doxorubicin.

Despite thermosensitive liposome delivery gives higher peak values for both free and bound extracellular concentrations of doxorubicin in normal tissues, the concentration level is still lower than the half maximal (50%) inhibitory concentration (IC) of doxorubicin in normal tissue, which is 4.13 × 10^−5^ kg/m^3^ [47]. However, the rate of cell killing is found to be related to the area under the extracellular concentration curve (AUC_*e*_) [48, 49]. A simplified model in literature [49] shows that the logarithmic value of cell survival fraction is proportional to the AUC_*e*_. Values for AUC_*e*_ under 2-hour infusion and thermosensitive liposome delivery are compared in Table 4 which shows that the 2-hour infusion leads to high AUC_*e*_ in the first 48 hours of the treatment, suggesting that 2-hour direct infusion of doxorubicin is likely to cause more cell death in normal tissues than thermosensitive liposome delivery. 

**Figure 9:**
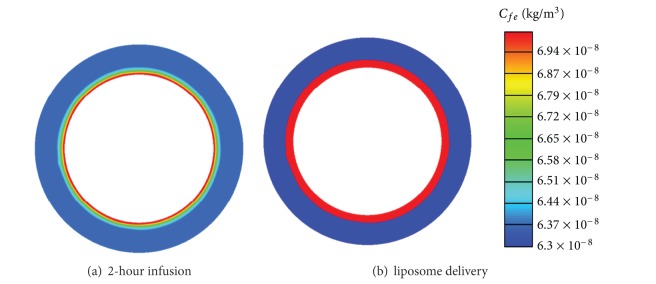
Spatial distribution of free doxorubicin extracellular concentration in normal tissues at 25-hour with 2-hour infusion and liposome delivery (dose = 50 mg/m^2^).

Because heating can be controlled and localised in tumour, the temperature in normal tissues would be lower than the hyperthermia temperature required for the release of doxorubicin from liposomes. During the heating period, doxorubicin enters normal tissue only by diffusion and convection from tumour. This leads to doxorubicin being mainly concentrated in the region surrounding the tumour, as shown in Figure 9(b). However, under 2-hour direct infusion, doxorubicin is carried by blood into normal tissues. This leads to doxorubicin concentration reaching a higher level in the entire region of normal tissues, shown in Figure 9(a). Hence, thermosensitive liposome-mediated drug delivery performs better in reducing drug concentration in the main region of normal tissues, which may help lower the risks of associated side effects. 

**Figure 10:**
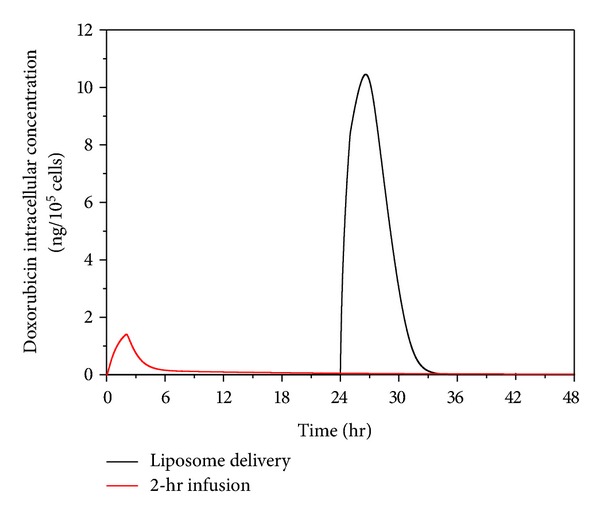
Doxorubicin intracellular concentration as a function of time, for thermosensitive liposome delivery and 2-hour direct infusion (dose = 50 mg/m^2^).

Figure 10 presents the intracellular doxorubicin concentration in tumour for thermosensitive liposome delivery and 2-hour direct infusion. The intracellular concentration under 2-hour direct infusion displays a quick rise after drug administration until it reaches a peak and then decreases. The intracellular concentration under thermosensitive liposome delivery remains at zero until 24 hours, but there is a sharp rise to a high peak immediately after heating. However, as heating ceases and tumour tissue cools down to the physiological temperature range, no new doxorubicin is released and the intracellular concentration drops rapidly to a low level. Compared with 2-hour direct infusion, the thermosensitive liposome delivery leads to a much higher peak intracellular concentration.

Compared with the study reported by El-Kareh and Secomb [12], lower free doxorubicin extracellular and intracellular concentrations are found here. This is because the present model accounts for the effect of binding between doxorubicin and proteins in plasma. Since 75% doxorubicin is bound with proteins, less free doxorubicin is available in plasma for crossing the vasculature wall and entering the interstitial space, which leads to less drug uptake by tumour cells. Together with the experimental evidence [13], our predictions demonstrate that protein binding of anticancer drugs in plasma is an important factor that should be included in future mathematical models.

**Figure 11:**
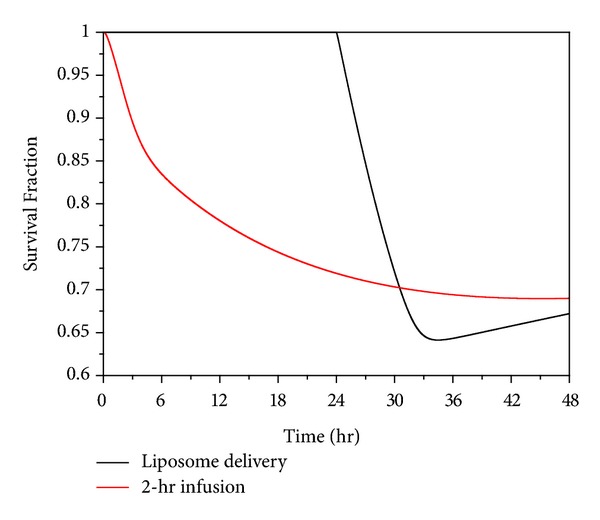
Temporal profiles of predicated tumour cell survival under 2-hour direct infusion and thermosensitive liposome delivery (dose = 50 mg/m^2^).

Figure 11 shows the fraction of survival cells by applying the pharmacodynamics model described by (29). As can be observed, the therapeutic effectiveness of 2-hour direct infusion can last for a longer period after administration. Fewer tumour cells are killed after 36 hours because the intracellular concentration is below the threshold for cell killing (Figure 10). On the other hand, the effect of thermosensitive liposome delivery takes place after the start of heating. Highly effective tumour cell killing is observed since the intracellular concentration rises to a very high level in a short period of time (Figure 10). However, because temperature drops to the normal physiological range after heating, and no doxorubicin is released at this temperature, both the extra- and intracellular concentrations fall rapidly to a low level (Figures 7 and 10). Since the rate of cell killing caused by doxorubicin is slower than the rate of cell proliferation, the survival faction starts to rise after 34 hours. Nevertheless, thermosensitive liposome delivery leads to higher tumour cell death in a shorter time period than 2-hour direct infusion. On the other hand, the 2-hour direct infusion yields a higher extracellular concentration in normal tissues, which is undesirable as high drug concentration in normal tissue may increase the risk of side effects in patients.

Although the present numerical study offers some new insight into how anticancer treatment efficacy could be affected by different drug delivery modes, the mathematical models involve a number of assumptions. For example, realistic changes in tumour temperature during heating and after heating are ignored, and step changes are specified instead. In clinical practice, tumour temperature profiles (temperature versus time curve) may vary depending on the heating method applied. Moreover, temperature distribution in tumour tissue is likely to be nonuniform. These factors can influence the outcome of anticancer treatments. Other assumptions include an idealised geometry for the tumour and normal tissues, uniform transport properties, and a uniform distribution of microvasculature for administration of anticancer drug.

## 4. Conclusion

Doxorubicin delivery into solid tumour by direct continuous infusion and thermosensitive liposome are studied by mathematical modelling, and the anticancer effectiveness is evaluated in terms of the survival fraction of tumour cells. Our computational results show that thermosensitive liposome-mediated delivery offers a lower drug concentration in normal tissues than direct infusion of nonencapsulated doxorubicin, which may help reduce the risk of associated side effects. In addition, thermosensitive liposome delivery achieves a significantly higher peak intracellular concentration, and hence more rapid and effective tumour cell killing in a short time period of treatment.

## Figures and Tables

**Table 1:** Parameters for tumour and normal tissues (symbols are defined near the equations in which they first appear).

Parameter	Unit	Tumour Tissue	Normal Tissue	Reference
*S*/*V*	m^−1^	20000	7000	[15–18]
*K*_*v*_	m/Pa·s	2.10 × 10^−11^	2.70 × 10^−12^	[15–18]
*K*	m^2^/Pa·s	3.10 × 10^−14^	6.40 × 10^−15^	[15–18]
*ρ*	kg/m^3^	1000	1000	[18]
*μ*	kg/m·s	0.00078	0.00078	[18]
1/*κ*	m^−2^	4.56 × 10^16^	2.21 × 10^17^	[15–18]
*p*_*v*_	Pa	2080	2080	[15–18]
*π*_*v*_	Pa	2666	2666	[15–18]
*π*_*i*_	Pa	2000	1333	[15–18]
*σ*_*T*_		0.82	0.91	[15–18]
*K*_ly_ *S*_ly_/*V*	(Pa·s)^−1^	0	4.17 × 10^−7^	[18]
*p*_ly_	Pa	0	0	[18]
*D*_*c*_	10^5^ cell/m^3^	1 × 10^10^	1 × 10^10^	[12, 22]
*φ*		0.4	—	[12, 22]

**Table 2:** Parameters for liposome (symbols are defined near the equations in which they first appear).

Parameter	Unit	In tumour	In normal Tissue	Reference
*P*_*o*_	m/s	3.42 × 10^−9^	8.50 × 10^−10^	[23, 24]
*h*		71	—	—
*D*	m^2^/s	9.0 × 10^−12^	5.8 × 10^−12^	[20, 23]
*σ*_*l*_		0.95	1.0	—
*A*_1_	kg/m^3^	6.90 × 10^−3^	6.90 × 10^−3^	[25]
*A*_2_	kg/m^3^	8.37 × 10^−5^	8.37 × 10^−5^	[25]
*k*_1_	s^−1^	1.22 × 10^−2^	1.22 × 10^−2^	[25]
*k*_2_	s^−1^	4.17 × 10^−6^	4.17 × 10^−6^	[25]
*t*_*h*_	hr	24	—	—
*t*_*d*_	s	3600	—	—
*k*_*rel*⁡_37°C_	s^−1^	0	—	—
*k*_*rel*⁡_42°C_	s^−1^	0.0078	—	—

**Table 3:** Parameters for doxorubicin (symbols are defined near the equations in which they first appear).

Parameter	Unit	Free doxorubicin	Bound doxorubicin	Reference
*P*_Tumour_*o*_	m/s	3.00 × 10^−6^	7.80 × 10^−9^	[18, 26]
*h*		2.56	—	—
*P*_Normal_	m/s	3.75 × 10^−7^	2.50 × 10^−9^	[18, 26]
*D*_Tumour_	m^2^/s	3.40 × 10^−10^	8.89 × 10^−12 ^	[18, 22, 27–31]
*D*_Normal_	m^2^/s	1.58 × 10^−10^	4.17 × 10^−12^	[18, 22, 27–31]
MW	kg/mol	0.544	69.0	[12, 18]
*σ*_*d*_		0.15	0.82	[18, 32]
*k*_*a*_	s^−1^	0.833	—	[22]
*k*_*d*_	s^−1^	—	0.278	[22]
*V*_max⁡_	kg/10^5^ cells s	4.67 × 10^−15^	—	[22, 33]
*k*_*e*_	kg/m^3^	2.19 × 10^−4^	—	[22, 33]
*k*_*i*_	kg/m^3^	1.37 × 10^−12^	—	[22, 33]
*k*_max⁡_	s^−1^	1.67 × 10^−5^	—	[34]
EC_50_	kg/10^5^ cells	5 × 10^−13^	—	[34]
*D*	kg	8.56 × 10^−5^	—	[1]
*A*	m^−3^	74.6	74.6	[22, 35]
*B*	m^−3^	2.49	2.49	[22, 35]
*C*	m^−3^	0.552	0.552	[22, 35]
*α*	s^−1^	2.43 × 10^−3^	2.43 × 10^−3^	[22, 35]
*β*	s^−1^	2.83 × 10^−4^	2.83 × 10^−4^	[22, 35]
*γ*	s^−1^	1.18 × 10^−5^	1.18 × 10^−5^	[22, 35]
*k*_*p*_	s^−1^	3.0 × 10^−6^	—	[36]
*k*_*g*_	s^−1^	3.0 × 10^−16^	—	[36]
CL_tumour_	s^−1^	1.48 × 10^−5^	0	[35, 37–39]
CL_tumour_	s^−1^	2.43 × 10^−3^	0	[35, 37–39]

**Table 4:** AUC_*e*_ with various drug delivery modes in the first 48 hours.

	Free AUC_*e*_ (kgs/m^3^)	Bound AUC_*e*_ (kgs/m^3^)
Liposome delivery	1.59 × 10^−6^	5.19 × 10^−6^
2-hour infusion	2.30 × 10^−6^	6.91 × 10^−6^
